# Viruses in case series of tumors: Consistent presence in different cancers in the same subject

**DOI:** 10.1371/journal.pone.0172308

**Published:** 2017-03-03

**Authors:** Laila Sara Arroyo Mühr, Maria Hortlund, Zurab Bzhalava, Sara Nordqvist Kleppe, Davit Bzhalava, Emilie Hultin, Joakim Dillner

**Affiliations:** Division of Pathology, Department of Laboratory Medicine, Karolinska Institutet, Stockholm, Sweden; University of Alabama at Birmingham, UNITED STATES

## Abstract

Studies investigating presence of viruses in cancer often analyze case series of cancers, resulting in detection of many viruses that are not etiologically linked to the tumors where they are found. The incidence of virus-associated cancers is greatly increased in immunocompromised individuals. Non-melanoma skin cancer (NMSC) is also greatly increased and a variety of viruses have been detected in NMSC. As immunosuppressed patients often develop multiple independent NMSCs, we reasoned that viruses consistently present in independent tumors might be more likely to be involved in tumorigenesis. We sequenced 8 different NMSCs from 1 patient in comparison to 8 different NMSCs from 8 different patients. Among the latter, 12 different virus sequences were detected, but none in more than 1 tumor each. In contrast, the patient with multiple NMSCs had human papillomavirus type 15 and type 38 present in 6 out of 8 NMSCs.

## Introduction

The International Agency for Research on Cancer has classified >16% of all cancers in humans as caused by infections [1]. The virus-associated cancers have a greatly increased incidence in immunosuppressed individuals with an impaired ability to control virus infections, such as AIDS patients or solid organ transplant recipients [2–6]. The cancer that has the most highly increased incidence among the immunosuppressed is non-melanoma skin carcinoma (NMSC) [7–9], but does not have an established infectious etiology. A number of infections have been found in NMSC, with Human papillomavirus (HPV) being the most commonly found [10–15], but it is unclear whether any one of the infections detected so far are etiologically linked to the tumor.

Sequencing of all nucleic acids present in a sample is today a routine procedure [16–19]. The human sequences can be removed in the bioinformatics analysis of the sequences, leaving the metagenome (non-human genomes of e.g. bacteria and viruses) for study [20–22]. Virus detection that is dependent on prior knowledge of sequences, for example PCR with specific primer sets, can give a biased representation of which viruses that are present [23] and a simple sequencing of all nucleic acids in a sample gives a more unbiased representation of the viruses that are present.

Sequencing of the viral metagenome in skin samples reported that >95% of the virus sequences found belonged to the Papillomaviridae family, mostly to the β- and γ-genera [24], although recent improvements in bioinformatics using hidden Markov models now allow detection of a broader array of viruses in such samples [25]. A multitude of HPV types is present in skin lesions as well as on healthy skin, both among immunocompetent and immunosuppressed patients [11, 12, 26–29]. The description of which viruses that is most frequently present appears to be highly dependent on the different PCR primers used for HPV detection [12–15]. Sequencing of specimens without prior PCR is a more unbiased approach to detect viruses, even those which are not amplifiable by known PCR systems (2).

There are no fully adequate controls to use for case series of cancer tissue and it is therefore difficult to even guess if detection of a virus in a tumor suggests an etiological involvement or not. We reasoned that viruses consistently present in a series of independent tumors from the same patient might be more likely to be involved in tumorigenesis than viruses only found non-systematically in only some specimens. We therefore set out to test if there were indeed any virus types that were consistently found in such tumor series. Similar reasoning has been discussed before at a time point where HPV type-specific PCR-based approaches were used to detect HPV, however no single HPV type seemed to be consistently present in skin lesions of transplant recipients using biased PCR-based detection [30].

## Materials and methods

### Patients

We linked the 13429 patients having received a solid organ transplant in Sweden with the Swedish Cancer Registry and found 11127 tumors diagnosed >6 months after the transplantation. For this study, we selected 8 available diagnostic specimens from 8 different NMSCs (squamous cell carcinomas) that all had occurred in the same patient (diagnosed over a 10-year period, from 1993 to 2003). The localizations of the tumors were from “other and unspecified parts of the face” (6 specimens), “scalp and neck” (1 specimen) and “upper limb” (1 specimen). As controls, we identified 8 diagnostic blocks from NMSC (squamouns cell carcinomas) that had occurred in 8 different transplant recipients. The matching of “controls” to the case was performed according to 1) gender (male), 2) diagnosis (ICD7 code 191 “other malignant neoplasm of skin” including the forth digit “localization of the body”), 3) county of residence, 4) year of diagnosis (+/-10 years) and 5) age of the patient at diagnosis (+/-10 years).

The study was approved by the Ethical Review Board of the Stockholm region (Sweden) and has been conducted according to the principles expressed in the Declaration of Helsinki.

### Sample preparation

The FFPE blocks were sectioned at a quality-assured, certified laboratory (HistoCentre, Gothenburg, Sweden) where the process has been repeatedly validated to ensure uniform and contamination-free sectioning from blocks. Empty paraffin blocks were also sectioned in-between each sample to be used as negative controls.

Four 5μm sections of each formalin fixed paraffin embedded specimen (both tumor and blank paraffin blocks) were subjected to serial DNA extraction using the ExScale Automated DNA/RNA purification kit (art. no. ES-K110210FP) on a Magtration® 12GC UV at ExScale Biospecimen Solutions AB (Uppsala, Sweden), following the manufacturer’s instructions.

Analysis of the presence of the *hemoglobin subunit beta* gene with real-time PCR was performed to assess sample adequacy [31]. *Hemoglobin subunit beta* negative tumor specimens were re-analyzed to confirm negativity. If negativity was confirmed, specimens were excluded for further analysis.

All individual tumor blocks positive for *Hemoglobin subunit beta* together with 2 negative controls (1 water control and 1 blank paraffin control) were amplified with whole genome amplification (WGA) using the IllustraTM Ready-To-GoTM GenomiPhiTM DNA Amplification Kit (GE Health Care, United Kingdom). The manufacturer's instructions were followed, with these modifications: 2.5 μL of sample were diluted with 22.5 μL PCR-Grade water and 25 μL denaturation buffer. After incubation at 95°C for 3 min and cooling on ice, 50 μL of the samples were added to the Ready-To-Go GenomiPhi lyophilized cake. After a further incubation at 30°C for 7 h and inactivation at 65°C for 10 min, products were diluted in the ratio 1:2 in PCR-Grade water and quantified with QuantiFluor-ST (Promega, United States).

### Illumina sequencing

50 ng of genomic DNA per sample was tagmented with 2 unique indices, as described (Nextera DNA Sample Preparation kit, user guide revision B (Illumina)). The individual libraries were validated, normalized to 4 nM, pooled, denatured and diluted, resulting in a 2.6 pM DNA solution, which was spiked at 1% PhiX control and the library pool was sequenced paired-end 151+151 cycles using NextSeq 500 High Output reagent kit (Illumina) as described in the user guides “Denaturing and Diluting Libraries for the NextSeq 500” revision A and “NextSeq 500 kit Reference Guide” revision F.

### Bioinformatics

The index tags were used to assign the sequence reads to the individual samples. Sequences were trimmed according to their Phred quality scores [32] and then screened against the human reference genome hg19, as well as bacterial, phage and vector sequences (obtained from GenBank using BWA-MEM [33]) and reads with >95% identity over 75% of their length to human DNA were removed. The remaining sequences were normalized (http://ged.msu.edu/papers/2012-diginorm) and then assembled using the Trinity [34], SOAPdenovo-Trans (http://soap.genomics.org.cn/) and IDBA-UD [35] assemblers into contiguous sequences (contigs). Reads before assembly were re-mapped to assembled contigs. We used several assembly algorithms and re-mapping of all singleton reads to assembled contigs in order to validate assembly results [20, 36].

Taxonomic classification of assembled contigs used GenBank and paracel blast (www.paracel.com) to classify into 1) previously known sequences, 2) related to previously known sequences, or 3) unrelated to any previously known sequences. Papillomavirus-related contigs and singletons were checked to identify possible artifactual “chimeric” sequences (contigs containing sequences originating from different viruses), as described [20]. The sequence that aligned to its most closely related sequence in GenBank was divided into three equal segments. If at least one segment differed in similarity to the corresponding overlapping parts with more than 5% the sequence was considered a “possible chimera”.

Reads classified as “unrelated to any previously known sequences” were also analyzed with HMMER (the hmmsearch algorithm) [37] to search for distant homologs of already known viruses among the contigs. HHMER is designed to search sequence databases and identify remote homologues by implementing profile hidden Markov models. As reference database, we used the database constructed by Peter Skewes-Cox et al. (http://derisilab.ucsf.edu/software/vFam) [38], which includes viral profile hidden Markov models (“vFams”) from all the virally annotated proteins in RefSeq [38]. The HMMER algorithm calculates the E-value to order top sequences. We selected HHMER hits with e-value less than 1e-5. If sequences had hits with several different virus families, the hit with the lowest E-value was used.

Analyses used in-house R (www.R-project.org/), python (www.python.org) and bash (http://www.gnu.org/software/bash/) scripts running on a high performance (40 core, 2 TB RAM) Linux server. Specimens were classified as positive for viruses if at least 5 reads were detected.

## Results

Eight different NMSC from the case and 8 NMSC from the different patients were sequenced using the Illumina technology. There were ~970 million sequencing reads, from which <0.01% belonged to viral sequences (Table 1).

**Table 1:** Taxonomic assignment of sequencing reads.

	8 NMSCs^a^ same patient		8 NMSCs^a^ different patients		All	
	Reads	%	Reads	%	Reads	%
Bacteria	71196424	16.01	31188714	7.94	122696992	12.65
Human	353941360	79.6	342072130	87.13	754180186	77.74
Other	16171088	3.64	15083268	3.84	53159844	5.48
Unknown	3316424	0.75	4245870	1.08	40072660	4.13
Virus	38416	0.01	38	0.00	45970	0.00
TOTAL	444663712	100	392590020	100	970155652	100

The number of reads detected by taxonomic division in the cases of NMSCs from the same patient and in the 8 NMSCs from different patients, as well as the total number of reads in the sequencing run. The classification “Other” included reads that mapped to contigs with similarities to other sequences present in GenBank (e.g. plants, synthetic constructs, polymerase background reactivity). Reads mapped to contigs that did not give any hit to genomes deposited in GenBank were classified as “Unknown”. Reads detected in negative controls (water and blank paraffin block) as well as undetermined reads (reads failing the index identification) are only included in the “All” column.

^a^NMSCs: Non-melanoma skin carcinoma.

The Blast-based bioinformatics pipeline revealed 45970 viral reads, of which 38416 (83.6% of viral reads) were detected in the 8 different NMSC tumors belonging to the case and only 38 reads were detected in the 8 NMSC specimens belonging to the different patients (Table 2). Most viral reads (26308 viral reads) were from Human Herpesvirus 7 (HHV-7), which was found in only one NMSC specimen. Human papillomavirus (HPV) was detected in 6/8 NMSC specimens retrieved from the case patient (all 6 of them had a double infection with both HPV 15 and HPV 38). Only one NMSC among the controls had HPV detected (a single HPV 38 infection). The same “Faustovirus-related” apparently environmental virus was found in both case specimens, control specimen as well as in the water control with 2348, 20 and 5562 reads, respectively (Table 2).

**Table 2:** Classification of viral reads.

	8 NMSCs^a^ same patient		8 NMSCs^a^ different patients		Water	Paraffin blank block	All
Virus family	Reads	Pos^b^ Specimens	Reads	Pos^b^ Specimens	Reads	Reads	Reads
Papillomaviridae^B^	9760	6/8	18	1/8	0	0	10096
HPV-15	8424	6/8	4	0/8	0	0	8698
HPV-38	1336	6/8	14	1/8	0	0	1398
Herpesviridae^B^	26308	1/8	0	0/8	0	0	27584
HHV-7^B^	26308	1/8	0	0/8	0	0	27584
Related to Faustovirus^B^	2348	2/8	20	1/8	5652	0	8290
Baculoviridae^H^	4258	2/8	6134	1/8	0	0	10790
SE-882, 884, 890, 898	0	0/8	6134	1/8	0	0	6356
SE-896	3568	1/8	0	0/8	0	0	3722
SE-899	690	2/8	0	0/8	0	0	712
Marseilleviridae^H^	0	0/8	2210	1/8	0	0	2292
SE-895	0	0/8	2210	1/8	0	0	2292
Mimiviridae^H^	618	1/8	882	2/8	254	758	2606
SE-883, 893, 897	6	0/8	880	2/8	152	66	1152
SE-894	612	1/8	0	0/8	0	0	632
SE-885, 886, 888, 900, 902	0	0/8	2	0/8	102	692	822
Phycodnaviridae^H^	0	0/8	26	1/8	0	832	884
SE-881	0	0/8	0	0/8	0	832	854
SE-889	0	0/8	26	1/8	0	0	30
Unassigned^H^	8950	3/8	1056	1/8	36	0	10376
SE-887, 901	0	0/8	1056	1/8	0	0	1094
SE-891	32	1/8	0	0/8	0	0	32
SE-871	8918	2/8	0	0/8	0	0	9214
SE-892	0	0/8	0	0/8	36	0	36
Virus^B^	38416	7/8	38	1/8	5652	0	45970
Virus^H^	13826	5/8	10308	5/8	290	1590	26948
Total virus	52242	8/8	10346	6/8	5942	1590	72918

Description of viral sequences detected using paracel blast (^B^) and HMMER (^H^) bioinformatics algorithms. Unassigned^H^ classification corresponds to clusters which are constructed from viral sequences that do not share one viral family or do not belong to any viral family. Undetermined reads (reads failing the index identification) are only included in the “All” column.

^a^NMSC: Non-melanoma skin carcinoma

^b^Pos: Positive.

All reads that mapped to contigs that did not give any hit to genomes deposited in GenBank were classified as “unknown”, representing 4.13% of total reads (Table 1). Searching for distant homologs of already known viruses among these contigs using profile hidden Markov models revealed 23 (previously not described) viral contigs (designated as SE-sequences) related to up to 5 viral clusters (Table 2). None of the 23 SE-sequences were detected in more than 2 specimens each and only 2/5 clusters (Baculoviridae and Marseilleviridae) were not detected in the negative controls. The DNA viral contigs detected with the HHMER analysis (designatedSE881-SE902, SE871) have been deposited in GenBank (accession numbers KY006985-KY007007) and the deep sequencing data is deposited at NIH Sequence Read Archive (BioSample accessions SAMN06275823-SAMN06275840).

## Discussion

We report that sequencing of tumor tissues from different cancers occurring up to 10 years apart in the same individual, to a large extent tended to detect the same viruses present in multiple different tumors. While this finding does in itself not imply causality, it does suggest that viruses that are systematically detected in multiple tumors from the same patient might be more likely to be involved in tumorigenesis than viruses only found non-systematically in only some specimens.

The study is based on only a few patients and more patients would need to be sequenced to obtain more large-scale data. Although deep sequencing is today a routine, it is still costly and more large-scale studies would probably need to await less expensive technologies to be enabled. Another limitation is that the study compared only different cases of SCC, whereas benign tissues were not analyzed. As the study design was based on specimens from pathology archives, only tumors (and not benign tissues) were available.

The majority of viral reads detected (37.8% of total viral reads), were from HHV-7, a beta-herpesvirus known to infect almost all children by the age of three years with subsequent lifelong persistence, but all the HHV-7 reads were from a single specimen. The use of sequencing for virus detection is not dependent on prior knowledge of sequences, and therefore a more unbiased representation of viruses will be detected. However, the fact that we before sequencing used whole genome amplification (WGA) to increase the amount of DNA in the sample means that the number of reads obtained is not directly correlated to the amount of viral populations present [39]. This phenomenon is well described and is probably attributable to preferential amplification of genomic regions during initial WGA priming events [16, 40, 41]. The significance of the large amount of HHV-7 reads in a single specimen is therefore unclear.

Considering presence in multiple tumors, HPV was the most common virus found. Double infection of HPV 15 and 38 was detected in 6/8 NMSC belonging to the same patient, whereas the NMSCs from 8 different patients were HPV-positive only in 1/8 tumors (a single infection with HPV38). Both HPV 15 and 38 belong to the genus Beta-papillomavirus and are commonly found in patients with epidermodysplasia verruciformis. Further studies are needed to resolve the biological significance of the finding of HPV15 and 38 in multiple tumors from the same patient, but it can be concluded that as these cancers occurred up to 10 years apart the double infection with HPV15 and 38 must have persisted for many years in this subject. Persistence of HPV over time is known to be of importance for tumor maintenance and progression.

A large part of the sequencing reads from *de novo* sequencing projects are classified as “Unknown” due to incompleteness of public sequence databases or drawbacks of next generation sequencing technologies such as short read lengths and sequencing errors [20, 21]. The Blast-based bioinformatics pipeline revealed ~45000 viral reads, while the number of “Unknown” reads reached ~4M. Therefore, the more sensitive HHMER algorithms were used in order to search for distant homologs of already known viruses among the contigs classified as “Unknown” [25]. HHMER-based analysis identified 23 previously unknown viral contigs with up to 26948 viral reads. Each contig within a cluster was assigned a different SE-number (e.g: SE898) if its sequence did not overlap with other contigs related to the same cluster. Although the different SE-sequences were not overlapping, it is still possible that different contigs from the same cluster may belong to the same virus.

Viral sequences belonging to Mimiviridae, Phycodnaviridae, “related to Faustovirus”, and “Unassigned” (distantly related to viruses previously described, but not given any taxonomic designation yet), were detected in not more than 2 specimens each as well as in the water and/or the blank paraffin block used as negative controls. These sequences might therefore represent environmental deposit, highlighting the importance of including negative controls whenever performing metagenomic sequencing.

## Conclusions

In this study, deep sequencing was used for unbiased virus detection in NMSC specimens. Consecutive specimens from the same patient over several years and from different locations identified presence of HPV (types 15 and 38) in most of the specimens (6 out of 8), whereas only 1 out of 8 specimens from different patients was HPV-positive (type 38).
